# Emerging Roles for Platelets in Inflammation and Disease

**DOI:** 10.4172/2332-0877.1000149

**Published:** 2014-06-24

**Authors:** Yancy Ferrer-Acosta, Marieli González, Mónica Fernández, Washington A Valance

**Affiliations:** 1 University of Puerto Rico, Rio Piedras Campus, San Juan, Puerto Rico, USA; 2 Universidad Central del Caribe, Bayamón, Puerto Rico, USA; 3 University of Puerto Rico, Mayagüez Campus, Mayagüez, Puerto Rico, USA

## Abstract

Platelets and their interaction with cells of the immune system contribute through a variety of molecular mechanisms to support hemostasis and inflammation. These simple yet essential cells exert their effects in lymphocytes, monocytes, and neutrophils, both recruiting and modulating their function after activation. Emerging evidence is starting to define the mechanisms that allow platelets to also play pivotal roles in host defense. For example, platelet cell-surface expression of toll-like receptors allows platelets to direct neutrophil activation toward extracellular trap formation and facilitate the elimination of blood pathogens. In addition to these well-known receptors, two of the most recently discovered platelet receptors, C-type lectin receptor 2 (CLEC-2), and TREM-like transcript-1 (TLT-1), have been shown to modulate hemostatic and inflammation-related roles in platelets. This review will discuss the evolution of our understanding of platelet functions from hemostasis to inflammation, and highlight novel mechanisms that platelets use to mediate hemostasis under inflammatory pressure.

## Introduction

Hemostasis, thrombosis, and inflammation are mediated by platelets. Platelets are the specialized cells circulating in the blood that maintain blood vessel integrity and contribute to thrombosis; the other side of hemostasis [1]. Anyone who has taken advanced biochemistry has learned the coagulation cascade; unfortunately the purpose and beauty of the cascade, which is to generate thrombin and form a clot, is lost in the intricacy of these enzymatic reactions as they are taken out of context for class. Platelets regulate thrombin generation and in turn, subsequent platelet activation, fibrin production, clotting, hemostasis, and thrombosis. The platelet’s role in inflammation on the other hand, is less direct, but equally important.

Even though platelets may seem simple, they contribute to inflammation through the recruitment and activation of leukocytes [2,3]. Within the platelet’s bag of tricks are inflammatory mediators such as Platelet factor 4 (PF4), RANTES (Regulated on Activation, Normal T Cell Expressed and Secreted), IL1β (Interlukin1β), and CD40L (CD40 Ligand), which promote innate immune activation and modulation of the adaptive immune system [4–8]. In neutrophils, platelets stimulate fast responses such as the release of reactive oxygen species, myeloperoxidase, and proteases [2]. In monocytes, platelets induce intracellular signaling that leads to prothrombotic and inflammatory gene expression [9]. In lymphocytes, platelets mediate class-switching on B-cells and increase cytotoxic T-cell activity [10]. Secondary to hemostasis, platelet interactions with leukocytes may contribute to vascular injury and tissue damage in several inflammatory diseases including: atherosclerosis, inflammatory lung, bowel, and skin diseases, ischemic and inflammatory hepatitis, cancer, arthritis, glomerulonephritis and sepsis [11]. As demonstrated below, platelet-leukocyte interactions are critical to the clearance of bacterial, viral, and parasitic infections.

Emerging evidence is beginning to define the mechanisms that allow platelets to play a key role in host defense. Platelet expression of toll-like receptors (TLR2, TLR4 and TLR9) allows them to directly detect and eliminate blood pathogens, both by secreting microbiocidal molecules and by directly phagocytosing them [12,13]. These new emerging roles of platelets require a better understanding on how these cells modulate their different responses, and call for new investigations into platelet-specific molecules that fine-tune these responses that they use to keep the delicate balance between hemostasis and thrombosis. Current studies have outlined a new paradigm that suggests platelets mediate an immune-derived hemostasis or an immunohemostasis, that elicits a different set of molecular programs than the classic tools used in basic hemostasis [14,15]. This review will summarize and expand on the involvement of platelets in acute and chronic inflammation and describe two promising candidate receptors, CLEC-2 and TREM-like transcript (TLT)-1, as targets to manipulate platelet function and as potential intervention during the inflammatory response.

## Platelets: A Classic View

The coagulation cascade is one of the mechanisms that transfers vascular insult into action through the generation of a serine protease called thrombin. There are two well-described routes that can activate the clotting cascade: the extrinsic and the intrinsic pathway. Endothelial damage exposes tissue factor to Factor VII and initiates the extrinsic arm of the clotting cascade. The intrinsic pathway is initiated by phospholipids and Factor XII. Each of these pathways leads to membrane bound complexes that generate thrombin. The mechanisms of this serine protease cascade of activation are beyond the scope of this review, but for a more detailed explanation of these processes see references [16,17]. It is important to mention that these reactions in the fluid phase are kinetically unfavorable, but once the complexes that generate thrombin attach to the platelet’s surface, the rate of thrombin generation increases tremendously. After its generation, thrombin activates platelets and cleaves fibrinogen to fibrin. Together with red blood cells, these activated platelets form the basis of a clot.

**Figure 1:**
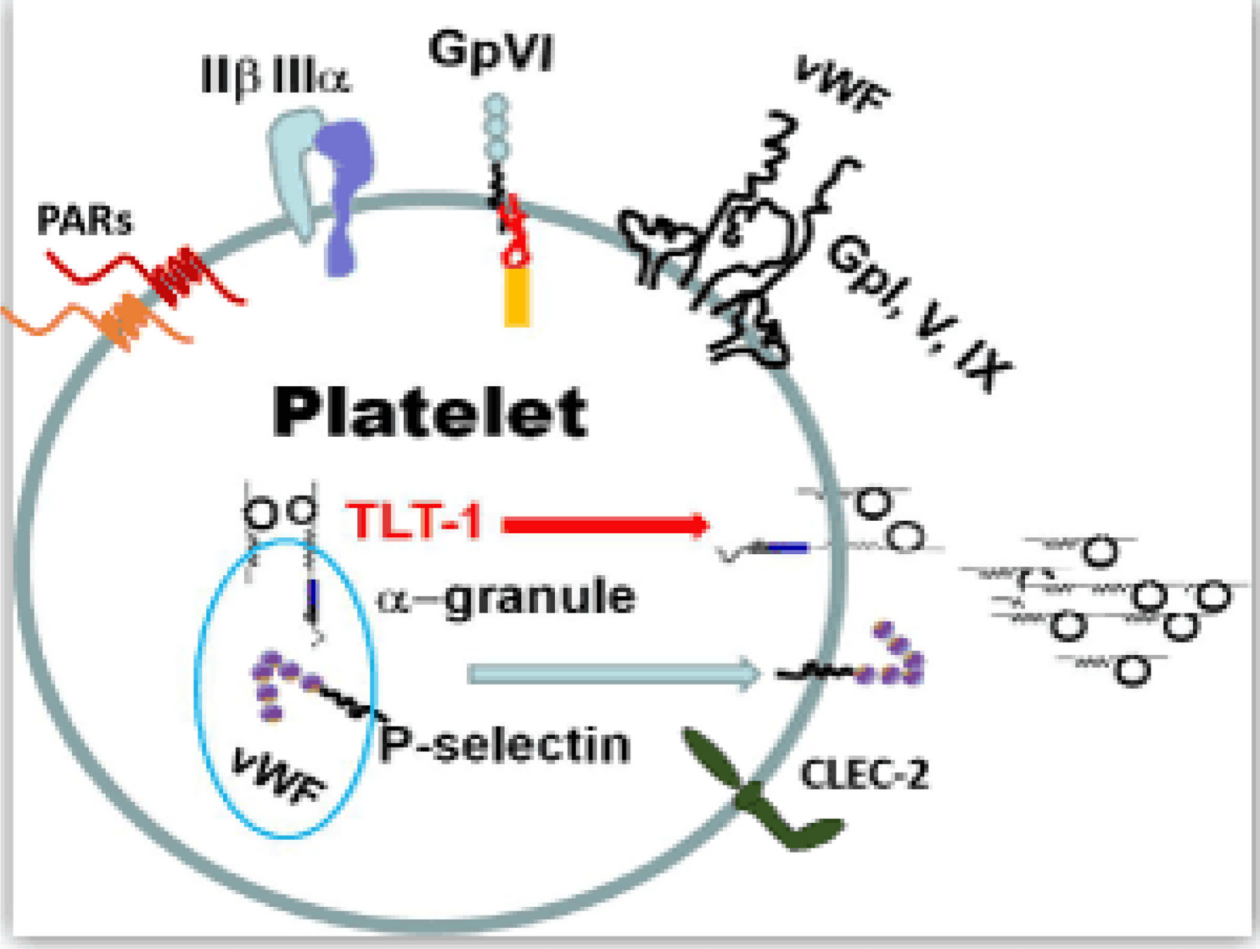
Molecules involved in mediating hemostasis and inflammation in platelets.

The vast majority of platelet functions can be defined by a handful of molecules held within the platelet’s bag of tricks (Figure 1). Platelets are activated by many agonists. The important endpoint of platelet activation is the acquired competence of the integrin receptor glycoprotein (GP) IIβ IIIα to bind fibrin [18]. Activated GP IIβ IIIα mediates platelet adhesion through a GP IIβ IIIα-fibrin-GP IIβ IIIα interaction. Thrombin, ADP, and thromboxane A2, are platelet activators that exert their effects through G-protein coupled receptors. Thrombin is arguably the most prolific of these activators because it acts upon two protease activated receptors (PARs 1 and 4) that are coupled to three different activating G-proteins: Gq, Gi, G12&13 [19–21]. Collagen, found in the extracellular matrix, also activates platelets, and it exerts its effects though an Fc-coupled receptor called GP VI (GPVI). In contrast to the PAR receptors, GPVI/Fc receptor mediates platelet activation signaling cascades through an immune tyrosine activating motif (ITAM) that recruits the tyrosine kinase SYK [22,23].

The platelet uses its bag of tricks to perform its multifunctional roles and relies on its receptors and granule contents. Among the most prominent molecules are the receptors that enable platelet activation (thrombin receptors-PAR 1 and 4 for thrombin; collagen receptor-GPVI), aggregation (the IIβ-IIIα fibrin-adhesion receptor) and adhesion (p-selectin; von Willebrand‘s Factor and its receptor GpI, V, IX) and recently described platelet receptors CLEC-2 and TLT-1.

Platelets activated by thrombin or bound to collagen, release their contents enclosed in their dense and α-granules, which expose more binding surfaces for coagulation factors such as prothrombin and factors V, VIII, IX&X promoting a positive feedback loop [24]. While the dense granules release small molecules such as ADP and serotonin, which aid in the coagulation process, the α-granules selectively release a much more complex mixture of mediators; mediators that we will refer to throughout the remainder of this review.

In platelets, GPIb-IX-V is an adhesion receptor complex that belongs to the leucine-rich repeat family. The major function of GPIb-IX-V complex is mediating the first step in platelet adhesion to the vessel wall. This complex binds to a collagen-tethered glycoprotein on the damaged sub-endothelial matrix, named von Willebrand Factor (vWF), to initiate thrombus formation at high-shear stress in flowing blood. High-shear results in increased GPIb-IX-V binding to vWF, which is rapidly secreted from platelet’s α-granules and endothelial cell’s Weibel-Palade bodies (storage granules) after tissue injury. Following binding of these proteins, platelets become activated. Platelet activation leads to shape change, spreading, and granule secretion, enabled by abrupt cytoskeletal rearrangements. This activation also promotes recruitment of additional platelets to the developing thrombus. The GPIb-IX-V complex can also bind to other platelet ligands such as thrombin, P-selectin, factor XI, factor XII, and high molecular weight kininogen [25,26].

## Platelets and Inflammation

Platelet α-granules harbor cytokines and membrane receptors that specifically mediate the inflammatory response. P-selectin (psel) is probably the most well studied α-granule resident protein. Used as a marker of platelet activation, psel binds to its ligand, Psel Glycoslated Ligand (PSGL-1), and initiates the platelet-endothelial and platelet-leukocyte interaction [27–31]. Integrin activation further stabilizes the platelet-leukocyte interaction. Thus, as leukocytes (monocytes and neutrophils) mediate the immune response, they have the added support of platelets as their “foot-soldiers”.

It is widely known that the presence of platelets increases immune function during in vitro immune studies. Immunology purists who study the innate immune response found that ethylenediaminetetraacetic acid (EDTA) is an important additive to their buffers [32]. EDTA chelates Ca++ and Mg++, inhibiting platelet activation and binding to leukocytes. Therefore, immune assays are often run without platelets and it is for these reasons that the platelet contribution to inflammation has gone unappreciated; that is, until recently.

The platelet-leukocyte interaction has shown to be more important than previously believed. In the spirit of “Retro”, classic studies are being revisited with a new twist: platelet depletion. Surprisingly, viral studies have shown that platelet depletion not only decreases the inflammatory response, but also the body’s ability to clear infection. Using two different models of viral infection in the liver, Iannacone et al. [33] demonstrated that platelet depletion lowered the inflammatory effects, but failed to clear the viral load. Re-introduction of platelets increased inflammation, but more importantly, resolved the viral infection. Apparently, platelets were important for the recruitment of cytotoxic T-cells to the site of infection. In a model using the mouse lymphocytic choriomeningitis virus (LCMV), it was found that platelet depletion changed both cytokine output and splenic organization, which led to systemic bleeding and death [34]. There are two series of studies that give insight into the mechanism behind the changes in T-cell function. The first begins with a study that establishes that platelets are the main source for CD40L in the blood [6]. An ensuing study investigated how platelets from wild type mice rescue the phenotype of CD40L null mice [10]. The authors convincingly show that CD40L on platelets enhances T-cell cytotoxicity and also mediates class-switching in B-cells. The second study shows that platelets have the ability to present antigen to T-cells in the context of class 1 [35]. These authors demonstrate that activated platelets exposed to antigen, presented the antigen to T-cells and promoted clearance of the parasite Plasmodium berghei, leading to 100% survival of the mice. On the other hand, B2 microglobin null platelets, under the same conditions, failed to raise a T-cell response, clear the infection, and all mice succumbed to the Plasmodium infiltration. From these studies it is clear that platelets are critical to the adaptive immune response and clearance of infectious disease.

Neutrophil endothelial traps or NETs, are a relatively new phenomenon by where activated neutrophils spew out their DNA to form intravascular traps during bacterial infection. NETs trap bacteria to coalesce them for more efficient removal [36]. They are found primarily in the pulmonary capillaries and liver sinusoids. NETs are visualized using fluorescent microscopy with stains for DNA (such as DAPI) and antibodies against histones. Clark et al. demonstrated that NETs are triggered by expression of TLR4 on platelets [37]. Platelet depletion greatly reduces NET presence and their ability to sequester bacteria [38]. Interestingly, NETs are remnant of evolutionary mechanisms found in invertebrates that used coagulation as a method to sequester foreign invaders [39].

## Emerging Roles

An emerging realization is that platelets use ulterior mechanisms than described by the handful of molecules to mediate hemostasis derived by inflammatory means. In an eloquent series of experiments it was shown that immune challenges in the absence of platelets causes bleeding, which in itself is not surprising [14]. The authors used the reverse arthus reaction as a model. This reaction is similar to the tuberculin test, where antigen is applied subcutaneously. If there are specific antibodies to recognize the antigen, leukocytes (mainly neutrophils) infiltrate the area causing edema and inflammation in reaction to the immune complexes at the site of injection. In a search to identify the hemostatic mechanism responsible for the bleeding, they performed the arthus reaction on mice deficient for many of the molecules used to define our understanding of hemostasis (B3, vWF, GPIV, psel, CaldagGEF and GP1b: Figure 1). Surprisingly, each of these mice maintained hemostasis, suggesting that the mechanisms used to clear infectious diseases initiate a differential hemostatic program. We finish our review on an emerging role of platelet function, and introduce two candidate receptors that may provide answers to this conundrum: CLEC-2 and TLT-1.

## C-type lectin receptor 2 (CLEC-2)

It is only recently that platelets are beginning to be recognized as critical players of processes beyond their classical role in hemostasis. In this sense, a novel platelet receptor has been described with new unexpected roles. The C-type lectin receptor 2 (CLEC-2) is a newly identified type II membrane receptor with high mRNA levels found in megakaryocytes [40]. In addition to platelets, CLEC-2 is expressed in a variety of cells including monocytes, dendritic cells and granulocytes [41], opening the possibility of many yet to be identified roles.

There are several variations of CLEC-2. In mice, CLEC-2 splice variants lacking either exon(s) 2 or 2/4 have been identified [42]. These studies demonstrate that the transmembrane domain, coded by exon 2, may regulate its retention and localization in the cytoplasm. The full-length receptor is cleaved by a protease and releases a soluble form. The soluble form has been described by two groups in humans suggesting a potential conserved role for these fragments [42,43]. Although it has been shown that aprotinin and PMA inhibits CLEC-2 cleavage, the exact protease has not been identified nor has its significance of these ulterior forms of CLEC-2 been revealed in platelet function [42].

The hunt for the ligand of the snake venom toxin rhodocytin (also called aggretin), led to the discovery of the CLEC-2 receptor [44]. Rhodocytin, a C-type lectin snake venom toxin similar to convulxin, induces robust platelet aggregation by inducing CLEC-2 tyrosine phosphorylation of its cytosolic tail and downstream activation of PLCγ2 [44,45].

Recently, CLEC-2 has been found to bind Fucoidan, a sulfated polysaccharide derived from the brown seaweed Fucus vesiculosus [46]. It is awkwardly called a “non-anticoagulant” because it does not exhibit the anticoagulant properties of the sulfated polysaccharide heparin [47]. Fucoidan decreases bleeding time in mice and humans with hemophilia and thus holds promise as an intervention for this bleeding disorder [47]. The original mechanism of Fucoidan was thought to be due to inhibition of tissue factor pathway inhibitor (TFPI). However, in subsequent studies using CLEC-2 null mice it was shown that Fuciodan induces platelet aggregation in wild type mice but not in CLEC-2 null mice demonstrating that Fuciodan activates platelets through the receptor CLEC-2 [46]. While Fucoidan’s binding of CLEC-2 does not exclude any effects it may have on TFPI, these studies do outline a potential role for CLEC-2 in the treatment of hemophilia.

Early studies on CLEC-2 highlighted its similarities to the platelet collagen receptor GPVI, which has an established role in clot formation by mediating stable platelet adhesion and aggregation in response to collagen [48]. However, subsequent investigations determined that although GPIV and CLEC-2 regulate Syk phosphorylation via ITAM and hemITAM sequences respectively, they have unrelated physiological ligands and distinct roles [49–51]. The physiological ligand for CLEC-2, podoplanin (PDPN), is expressed in a variety of tissues, but not on vascular endothelial cells [52–54]. The absence of PDPN in the vessel wall strongly suggests that unlike GPVI, CLEC-2’s role might not be primary hemostasis. In fact, there is conflictive evidence about whether CLEC-2 plays a significant role in hemostasis and thrombosis. While antibodies against platelet CLEC-2 have no significant effect on platelet aggregation to other agonists, CLEC-2-depleted platelets showed reduced platelet aggregation to collagen and reduced thrombus formation in mice after a ferric chloride injury model [55]. However, reduced collagen-dependent platelet aggregation in vitro was not reproducible in a separate CLEC-2 deficient mouse model, putting in question CLEC-2’s function in vivo [56].

Recent evidence supports a role for CLEC-2 in the separation of blood and lymphatic vasculature [57–59]. The importance of the interaction between CLEC-2 and PDPN was established when PDPN null mice bled from the high endothelial venules (HEVs) after immunization [45,60,61]. HEVs are specialized blood vessels that permit leukocyte infiltration into the lymph nodes for immune surveillance. PDPN as well as CLEC-2 deficient mice, showed spontaneous bleeding in both the mucosal lymph nodes and the draining peripheral lymph nodes after immunization. The loss of vessel integrity was explained by a reduced release of sphingosine-1-phosphate from CLEC-2 or PDPN null mice, leading to reduced induction of vascular endothelium cadherin (VE-cadherin) in surrounding fibroblastic reticular cells. VE-cadherin or vascular endothelium cadherin, is a calcium-dependent cell-cell adhesion glycoprotein that regulates the integrity and permeability of the cellular junctions.

Subsequent studies supported a role for the CLEC-2/PDPN axis in the regulation of a newly described form of hemostasis termed: lymphovenous hemostasis. Under conditions of impaired lymphovenous valve function, which prevents the blood’s blackflow into the lymphatic system, platelet CLEC-2/PDPN interaction can stabilize thrombi in the lymphatic endothelium to prevent retrograde blood flow [62]. Furthermore, in inflammation-induced hemorrhage, mice deficient in CLEC-2 bled when challenged with the arthus reaction, pointing to a role for CLEC-2 and ITAM-dependent signaling in immune-derived bleeding [63].

Taken together, strong evidence points towards a role of platelet immune-like receptors in settings outside primary hemostasis. These new realizations may have potential implications in our understanding and treatment of diseases such as sepsis and dengue, where immune derived bleeding may have a negative impact on clinical outcome.

The Triggering Receptor Expressed on Myeloid cells-like transcript-1 (TLT-1)

TLT-1 was first characterized as a putative inhibitory receptor because it contains an Immunoreceptor Tyrosine-based Inhibitory Motif (ITIM) in its cytoplasmic domain and it was hypothesized to inhibit other TREM family members [64–67]. Its role in hemostasis is much less defined than CLEC-2.

**Figure 2:**
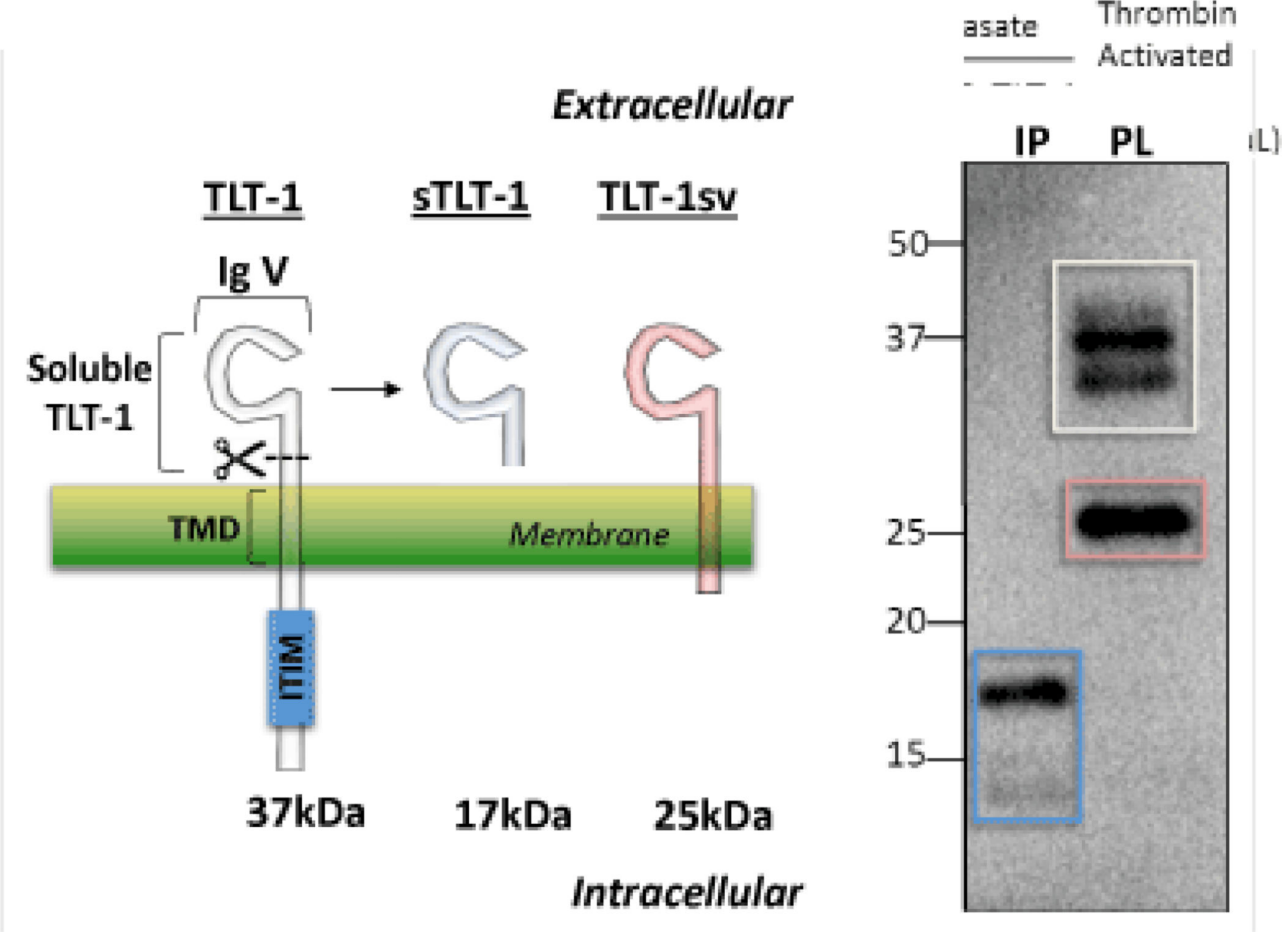
TREM-like transcript (TLT)-1 isoforms in platelets.

Four TLT-1 isoforms have been isolated to date (Figure 2). In platelets, three isoforms have been reported: TLT-1 full length (TLT-1) and TLT-1 splice variant (TLT-1sv); the third is the soluble isoform that is released upon platelet activation [64,66,68]. This third soluble isoform corresponds to the extracellular sequence of the protein. The fourth isoform (TLT-1s) has only been identified in pre-osteoclasts and contains a very short extracellular domain fragment, the transmembrane and the intracellular domain [69]. Although the full length TLT-1 has been shown to bind SHP-1 and SHP-2 in model cell lines, to date, no inhibitory functions have been attributed to this motif in platelets [64,66]. Full length TLT-1 is a 34 kDa Ig domain-containing receptor expressed in megakaryocytes and platelets. Although splice variants have been identified in other cell types [69], the extracellular Ig domain-containing form has only been found in platelets (Figure 2). Our discussion will focus on the platelet isoforms. Interestingly, sTLT-1 is the platelet’s fourth most abundantly released molecule and has been detected in the serum but not in plasma of healthy mice and humans [68,70].

Left panel, Representation of the two isoforms reported for TLT-1 on platelets and the cleaved soluble fragment generated upon platelet activation. These proteins consist of a common extracellular immunoglobulin V domain (IgV). The full length form of TLT-1 (TLT-1, white, 37 kDa) and isoform TLT-1sv (red, 25 kDa) both contain a transmembrane domain (TMD) but TLT-1 harbors an immunoreceptor tyrosine-based inhibition motif (ITIM). Soluble TLT-1 (sTLT-1, blue, 17 kDa) which is released from platelets upon activation. Right panel, In lane 1 (IP) immunoprecipitation followed by western blot of sTLT-1 from platelet releasate after activation with thrombin (0.01 U/mL). In lane 2, TLT-1 and TLT-1sv isoforms from platelet lysate. The two bands observed in TLT-1 (white square) suggest posttranslational modifications of TLT-1.

The first evidence that TLT-1 plays a role in the hemostatic mechanism of platelets was identified with the use of a single chain fragment (scFv) called C10 [71]. TLT-1 specific antibodies were isolated from a scFv library of which C10 showed the greatest binding affinity. In aggregation assays, C10 demonstrated a dose-depended inhibition of platelet activation with low-dose thrombin activated platelets. Higher doses of thrombin overcame C10 mediated inhibition. Consistent with these findings, platelets from treml1−/− mice also displayed reduced aggregation to ADP, collagen, and thrombin. TLT-1 null mice have increased tail-bleeding time when compared to controls and their platelets show decreased fibrinogen binding in vitro [72].

The soluble fragment of TLT-1 seems to play a distinct role in platelet function as well. Although the mechanism of sTLT-1 function remains to be elucidated, we have shown that sTLT-1 increases both platelet adherence and actin polymerization that appears to lead to enhanced platelet aggregation and contact with endothelial cells [73]. As pointed out above, sTLT-1 is not found in the plasma of healthy individuals, therefore when sTLT-1 levels are elevated in plasma it suggests both platelet activation and TLT-1 involvement, which urges further investigation into a potential role for TLT-1 in that particular disease. The first such evidence was identified in patients with sepsis.

## TLT-1 as possible modulator of Sepsis

Sepsis is a systemic inflammatory response caused by severe infection [74]. This disease can be accompanied by a low platelet count or thrombocytopenia, which can predict mortality in septic patients. Sepsis manifestation occurs when mechanisms meant to fight infection become unbalanced, leading to an acute state of inflammation. Severe sepsis may lead to septic shock, which results in decreased vascular pressure and multiple organ failure, leading to death. Disseminated Intravascular Coagulation (DIC), a condition often associated with sepsis, further increases mortality. In sepsis-associated DIC, platelets are systemically activated leading to increased fibrin deposition. D-dimers, which are fibrin degradation products, are used in the diagnosis of DIC. Dysregulation of clot formation and clot degradation results in consumption of coagulation factors, disruption of hemostasis, and can lead to simultaneous bleeding and clotting [75]. In these settings, inflammation and coagulation are intrinsically linked, and because of this, an understanding of how these factors are associated is needed to identify possible therapeutic targets.

Our early studies evaluated sTLT-1 levels in septic patients. A crosssectional study of 13 patients diagnosed with sepsis and compared to healthy individuals revealed a stark difference in the levels of sTLT-1 [72]. While healthy patients had virtually undetectable levels, patients with sepsis had levels that averaged over 300 ng/mL in plasma. A longitudinal study showed similar results and allowed a comparison with D-dimer levels [72]. Interestingly, sTLT-1 levels correlated with the presence of DIC better than D-dimers, suggesting sTLT-1 as a new candidate biomarker to test for DIC.

These findings prompted an investigation into a sepsis mouse model. Using a Lipopolysaccharide (LPS) model of sepsis we found that the treml1−/− mice succumb faster and in greater numbers than wild type mice. However, studies using the Shwartzman reaction, which mimics the mechanisms of DIC in a localized lesion, demonstrate that the treml1−/− mice bleed from the inflammatory insult, where the wild type mice do not. These results suggested that TLT-1 may be one of the mechanisms that platelets use to manage immune-derived hemostasis. Follow up studies using the treml1−/− mouse demonstrated that the treml1−/− mice are more susceptible to bacterial infection and that the use of a peptide derived from TLT-1, named LP17, can modulate the outcome to microbial infection in vivo; similar results were also achieved in a swine model [76,77]. These studies confirm that TLT-1 plays a role during the progression of bacterial sepsis, but moreover, suggest that it may be a therapeutic target.

Ongoing studies in the laboratory suggest that TLT-1 may modulate more than just platelet function. Additional potential targets for TLT-1 function are leukocytes and endothelial cells. It would be interesting to see if TLT-1 inhibitory antibodies such as C10 could induce the same outcomes as the LP17 peptide in the microbial studies. Our findings in studies of infectious disease may have long-reaching effects in cancer, atherosclerosis, and other inflammatory conditions.

**Figure 3:**
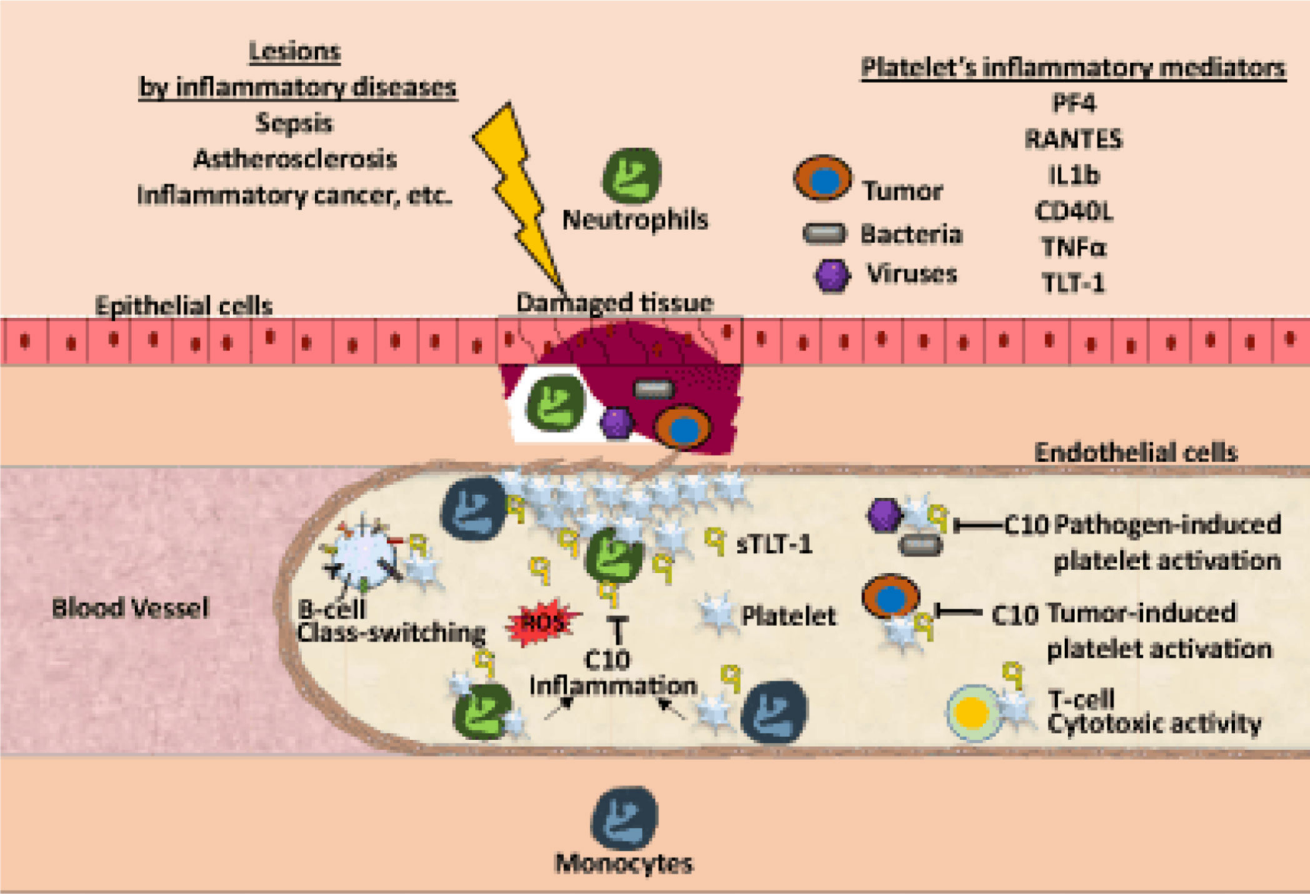
Schematic model of the role of platelets in inflammatory disease and a suggested view on how the C10 Anti-TLT-1 could be a therapeutic treatment in these diseases.

The generation of C10 [71], which specifically binds and inhibits sTLT-1 effect on thrombin-induced platelet activation, opens a new possible target to TLT-1 mediated contribution in inflammatory hemostasis (Figure 3). Thus, in conditions such as sepsis, this antibody holds promise as a new therapeutic treatment to modulate aberrant platelet activation. In addition to its potential in sepsis, our expanded view of diseases in which TLT-1 could play a role, suggest that in acute or chronic inflammatory conditions, modulation of platelet activation by C10 will provide a possible approach to control inflammation and hemostasis.

Platelets take on different roles upon the disruption of hemostasis and the promotion of inflammation in the vascular system. Platelets mediate B-cell class switching and increase T-cell cytotoxic activity. Once tissue damage is detected after infection or inflammation, platelets become activated at the site of damage and release factors that assist in their pro-inflammatory functions. Platelets recruit neutrophils and monocytes to the site of damage. While neutrophils release reactive oxygen species (ROS), monocytes promote the expression of pro-inflammatory gene expression. When regulation of this inflammatory response cannot be properly controlled by the endothelial cells this results in increased tissue damage. In the case of pathogen-induced platelet activation, this can cause a septic shock when platelet activation is excessive and hemostasis can be lost. In this review, we propose that C10 antibody can help diminish this excessive platelet activation through the blockade of soluble and platelet-bound TLT-1, modulating the inflammatory response and helping to restoring hemostasis.
